# Practical Considerations and Ethical Implications of Using Artificial Intelligence in Writing Scientific Manuscripts

**DOI:** 10.14309/crj.0000000000001629

**Published:** 2025-02-19

**Authors:** Muhammad Nadeem Yousaf

**Affiliations:** 1 Department of Medicine, Division of Gastroenterology and Hepatology, University of Missouri School of Medicine, Columbia, MO

The growing accessibility and sophistication of artificial intelligence (AI) tools have transformed many areas of research, including scientific writing. AI tools, such as natural language processing models and machine learning-based writing assistants, are increasingly used to help draft, edit, and refine scientific manuscripts. However, the use of AI in the writing process introduces both legal and ethical challenges. Various guidelines and policies have emerged, particularly from academic publishers, aimed at ensuring transparency and maintaining the integrity of scientific work. This editorial aimed to provide guidance for researchers on the ethical and practical considerations regarding the use of AI in writing scientific manuscripts, focusing on institutional policies, authorship accountability, intellectual property concerns, plagiarism issues, and image integrity.

## INSTITUTIONAL AND JOURNAL POLICIES: TRANSPARENCY AND GUIDELINES

As AI tools become more prevalent in scientific writing, academic institutions and journals are establishing clear guidelines regarding their use. The majority of well-known publishers provide guidance to authors, readers, reviewers, and editors concerning the role of AI-assisted technologies in the writing process, but industrywide standards have not yet been fully refined.^1^ The recently developed framework by American College of Gastroenterology (ACG) and Wolters Kluwer, the publisher of ACG journals emphasizes transparency and accountability, highlighting that although AI can assist in improving the readability and language of a manuscript, it should not replace the core tasks of authorship, such as generating scientific insights or drawing conclusions.^2^ ACG also requires authors to disclose the use of generative AI and AI-assisted technologies during the writing process. This disclosure fosters trust and ensures compliance with the terms of use of the AI tools. The goal is to provide clarity to readers, reviewers, and editors, helping them understand where and how AI was applied in manuscript preparation. Failure to disclose AI use can lead to ethical breaches, retractions, and potential damage to the professional reputations of both the researcher and the journal. The policy only covers the creation of new content and expressly forbids using AI on previously published material, preventing concerns around self-plagiarism or unauthorized content modification.

## AUTHORSHIP AND ACCOUNTABILITY: THE ROLE OF HUMAN OVERSIGHT

The rise of AI in scientific writing raises fundamental questions about authorship and responsibility. The ACG's authors instructions emphasize that authorship cannot be attributed to AI, as AI cannot take responsibility for the accuracy or integrity of scientific work. Authorship implies a set of ethical and intellectual responsibilities that only human researchers can fulfill. Every listed author must be accountable for the content of the manuscript, and AI cannot be assigned such responsibilities. Moreover, although AI can assist in writing, it cannot generate the intellectual contributions to scientific research. Scientific manuscripts must be the product of human insight and critical thinking. ACG's policy highlights the need for human oversight in the application of AI technologies, emphasizing that all AI-generated content should be thoroughly reviewed and edited by the authors to ensure accuracy, completeness, and lack of bias. The authors are ultimately responsible for ensuring that their work adheres to the highest standards of scientific integrity. This policy aligns with broader academic norms, such as the International Committee of Medical Journal Editors criteria for authorship, which require authors to have made significant intellectual contributions to the research and to be accountable for the final work. Failure to ensure human oversight and control can lead to inaccurate or misleading scientific conclusions, jeopardizing the validity of the research.

## INTELLECTUAL PROPERTY AND OWNERSHIP OF AI-GENERATED CONTENT

The use of AI in scientific writing introduces important questions about intellectual property (IP) ownership. Scientific manuscripts often contain novel ideas and discoveries, and researchers must be careful about the terms and conditions associated with the AI tools they use. Some AI platforms, such as OpenAI's Chat-GPT models, explicitly state that users retain ownership of the content generated through the tool. However, other platforms may have different terms, leading to potential conflicts over content ownership. ACG's policy ensures that authors remain responsible for the originality of their work, cautioning against the use of AI tools in ways that might lead to copyright violations or IP disputes. Because AI models are trained on vast data sets that may include copyrighted material, authors must be vigilant in ensuring that the AI-generated text does not unintentionally replicate existing works without proper attribution.

This highlights the importance of understanding the terms of service of the AI platform being used and adhering to proper citation practices.

## PLAGIARISM AND ETHICAL CONCERNS

Plagiarism is a serious ethical violation in academia, and the use of AI tools presents new challenges in this area. AI systems generate text based on large data sets, and although they aim to produce original content, there is always the risk of unintentional plagiarism if the AI-generated text closely resembles existing works.^3^ Academic journals including all of ACG's journals use plagiarism detection software to monitor submissions, and any AI-generated content that overlaps significantly with previously published works could be flagged as plagiarism. To address this, the authors should practice transparency and proper attribution when AI tools are used. The authors must disclose AI use to avoid accusations of misconduct and to ensure the originality of their work. In addition, because AI can generate authoritative-sounding but incorrect or biased information, researchers must carefully review the content produced by these tools, ensuring that it meets academic standards.

## THE GENERATION OF FABRICATED CITATIONS AND REFERENCES USING AI-ASSISTED TOOLS

One of the most pressing concerns in AI-assisted academic writing is the potential for generating fake citations and references^4,5^. This problem arises when AI tools produce fictitious or incorrect references that appear authentic but do not correspond to real sources. These generated references often mimic legitimate academic citations, complete with plausible journal titles, author names, and publication dates. However, on closer inspection, the cited works may not exist, or the citation details may be inaccurate, leading to false academic claims. The generation of fake citations by AI tools severely undermines the integrity of the peer-reviewed scientific process. Scholarly research depends on verifiable sources and accurate references that allow readers and reviewers to trace the intellectual lineage of ideas and verify the reliability of the claims being made. When citations are fabricated, this essential foundation of research collapses, leading to a cascade of misinformation. For instance, if one published article contains fake references and is later cited by others, the spread of false information becomes harder to detect and control, potentially polluting the knowledge base of a given field. The dangers of AI-generating fake citations extend beyond simple inaccuracies. Because AI models rely on large-scale data sets to produce content, they may unintentionally fabricate references by amalgamating parts of real sources with incorrect or fictional details. This creates the appearance of a legitimate scholarly foundation, making it difficult for readers and reviewers to identify problematic citations without extensive fact-checking. Researchers using AI tools must remain vigilant and ensure that all citations are factually accurate and correspond to real, verifiable sources. The academic community operates on a system of mutual trust, and violations such as the intentional use of fake citations can cause irreparable harm to the credibility of individual researchers and the broader field.

## PRACTICAL APPLICATION OF AI TO STREAMLINE RESEARCH WHILE PRESERVING ETHICAL STANDARDS

AI is transforming research, offering tools that assist with a variety of tasks, from systematic reviews to advanced data analysis.^6^ These AI tools have the potential to streamline many stages of the systematic review process, including developing and refining search strategies, screening titles and abstracts based on inclusion or exclusion criteria, extracting essential data from studies, and summarizing findings.^6^ AI can also efficiently scan vast databases to identify relevant articles and automatically organize metadata from diverse sources. Machine learning algorithms extend these capabilities by uncovering hidden patterns, trends, and correlations in complex datasets, enabling predictive analytics and intuitive visualizations that enrich research insights. However, these tools should not be seen as replacements for human expertise and judgment. Quality and ethical risks, such as biases in training data or inaccuracies in results, remain critical concerns. Researchers must view AI as a supplementary tool that optimizes processes while preserving the importance of critical quality checks, human evaluation, and meaningful intellectual contributions. Human oversight remains indispensable for interpreting findings, evaluating ethical implications, and ensuring the overall integrity of scientific reports. By balancing AI's efficiency with the irreplaceable depth of human analysis, researchers can leverage these technologies to enhance workflows, maintain ethical rigor, and drive meaningful scientific progress.

The integration of AI tools into the writing of scientific manuscripts presents both practical benefits and ethical challenges. Although AI can enhance readability and streamline the drafting process, it cannot replace the essential human contributions that define authorship, intellectual insight, and scientific integrity. Clear institutional and publisher policies should emphasize the need for transparency, accountability, and human oversight in the use of AI tools. The generation of fabricated citations and references, IP concerns, and risks related to image integrity highlight the ethical complexities posed by AI. To safeguard the credibility and trustworthiness of academic research, it is imperative that researchers carefully manage AI usage, ensure proper attribution, and maintain the originality of their work. As AI technology evolves, ongoing vigilance, refined policies, and adherence to ethical standards will be crucial in maintaining the integrity of scientific literature.

## DISCLOSURES

Author contributions: MN Yousaf wrote and edited the manuscript and is the article guarantor.

Acknowledgments: Neen LeMaster, Assistant Managing Editor of ACG Scholarly Publications. Neen helped in refining ACG policy in the editorial from managing standpoint.

Financial disclosure: None to report.

Informed consent was obtained for this case report.
